# Expression of angiotensin-converting enzyme 2, transmembrane serine protease 2, and sirtuin 1 proteins in lungs of different age groups

**DOI:** 10.36416/1806-3756/e20250127

**Published:** 2026-03-05

**Authors:** Ana Carolina Alves Lamounier, Francisca Carla Lucas Froio, Gabriel Ribeiro, Natália de Souza Xavier Costa, Jôse Mára de Brito, Luiz Vicente Ribeiro Ferreira da Silva-Filho, Thais Mauad

**Affiliations:** 1. Unidade de Pneumologia Pediátrica, Instituto da Criança e do Adolescente, Hospital das Clínicas, Faculdade de Medicina, Universidade de São Paulo - FMUSP - São Paulo (SP) Brasil.; 2. Departamento de Patologia, Faculdade de Medicina, Universidade de São Paulo - FMUSP - São Paulo (SP) Brasil.

**Keywords:** Angiotensin-converting enzyme 2, TMPRSS2 protein, human, Sirtuin 1, Lung, Aging

## Abstract

**Objective:**

Apart from the counter-regulation of angiotensin II levels in the renin-angiotensin system, angiotensin-converting enzyme 2 (ACE2) acts as a receptor for SARS-CoV-2, which is activated by transmembrane serine protease 2 (TMPRSS2) in target cells. Sirtuin 1 (SIRT1), an aging-related protein, controls ACE2 transcription in energy stress situations. This study aimed to evaluate the protein expression of ACE2, TMPRSS2, and SIRT1 in the lungs of children, adults, and elderly individuals.

**Methods:**

We used immunohistochemistry and software-assisted analysis to evaluate ACE2, TMPRSS2, and SIRT1 protein expression in autopsied lung tissue with minimal histological abnormalities and no clinical diagnosis of pulmonary disease or infection. The study population included 25 children (newborn to 19-year-old), 7 adults (20- to 59-year-old), and 11 elderly individuals (60- to 95-year-old). Of those 43 patients, 19 were female and 24 were male.

**Results:**

ACE2, TMPRSS2, and SIRT1 proteins were more expressed in the pulmonary parenchyma of children than in that of adults (p = 0.043, p = 0.008, and p = 0.032, respectively). SIRT1 expression was higher in the alveoli of children than in those of elderly patients (p = 0.008). No sex-based differences were observed. Spearman’s correlation coefficient showed that ACE2, TMPRSS2, and SIRT1 expression decreased with aging.

**Conclusions:**

ACE2, TMPRSS2, and SIRT1 were more expressed in the lung parenchyma (but not in the airways) of children than in that of older individuals. This could contribute to less severe COVID-19 lung disease in children.

## INTRODUCTION

In the renin-angiotensin system (RAS), angiotensin-converting enzyme 2 (ACE2) counteracts angiotensin II through its conversion to angiotensin1-7 [Ang (1-7)]. By activating the G protein-coupled Mas receptors, Ang (1-7) stimulates vasodilatory, anti-inflammatory, antioxidative, and antiproliferative effects.^1^ In animal studies, ACE2 protein lung expression reduces with senescence.^2^ ^,^ ^3^ In human tissues, ACE2 gene activity declines over the life course.^4^

ACE2 gained renewed attention during the COVID-19 pandemic because it is the main receptor of SARS-CoV-2. After binding to ACE2, SARS-CoV-2 is proteolytically activated by transmembrane serine protease 2 (TMPRSS2), thus facilitating viral entry into target cells, such as type II pneumocytes in the alveoli.^5^ ^-^ ^9^ Although TMPRSS2 function in human physiology is not well known, TMPRSS2 gene expression is stimulated by androgen hormones and is increased in androgen-dependent prostatic cancer.^10^

Differing expressions of ACE2 and TMPRSS2 proteins have been suggested to play a role in the difference in COVID-19 severity between pediatric and older patients.^11^ ^,^ ^12^ However, most studies have analyzed ACE2 and TMPRSS2 transcriptome and genome in lung samples from adults.^13^ ^-^ ^15^ In addition, there are scarce and contradictory data on the expression of ACE2 protein in the lungs of children. Schurink et al.^16^ and Silva et al.^17^ reported that pediatric lung samples had lower levels of ACE2, whereas Ortiz et al.^18^ and Zhang et al.^19^ showed that ACE2 protein was more expressed in the alveolar parenchyma of children than in that of adults and elderly individuals. 

An important protein linked to lung senescence is sirtuin 1 (SIRT1), which is known to be decreased in elderly individuals when compared with adults.^20^ SIRT1 is a histone deacetylase^21^ that plays a key role in cell survival. SIRT1 stimulates ACE2 transcription and protein expression under conditions of cell energy stress^22^ and could participate in the mechanisms leading to distinct ACE2 expression through the lifespan. 

In addition to the discrepant and scarce results regarding ACE2 and TMPRSS2 proteins expression in the lungs of children, there are no data on the expression of SIRT1 in the alveoli of children. Therefore, the present study sought to assess quantitatively ACE2, TMPRSS2, and SIRT1 protein expression in the pulmonary parenchyma and airways obtained by autopsy of individuals of a wide age range. Our results could provide insight into the intriguing differences in COVID-19 lung disease severity among age groups. 

## METHODS

### Study population

The present study was approved by the Brazilian National Research Ethics Committee (CAAE no. 80420917.4.0000.0065 and CAAE no. 30364720.0.0000.0068) and was performed in accordance with the Declaration of Helsinki. 

Lung samples were obtained from the autopsy archives of the Department of Pathology of the University of São Paulo School of Medicine, located in the city of São Paulo, Brazil. We selected lung tissue samples from the autopsies of 25 children who died between 1998 and 2020. The samples had minimal histological abnormalities and no clinical diagnosis of pulmonary disease or infection. Lung tissue samples from 7 adults (20- to 59-year age bracket) and 11 elderly individuals (60- to 95-year age bracket) whose lungs were normal on histology and who had no history of pulmonary disease were also selected. Adults and elderly patients were nonsmokers, the exception being one adult. 

In the group of children, the Brazilian National Research Ethics Committee granted us a waiver of written informed consent because of the impossibility of tracking down the families. We reviewed all available clinical charts and autopsy reports. The lungs of adults and elderly individuals were collected during the coroner’s autopsies, and there were no available clinical charts. A written interview was conducted, and the next of kin consented to donate tissue samples for research, as well as provided information related to the health status of the deceased adults and elderly individuals. 

### Tissue processing and immunohistochemistry

Each patient had 1-4 paraffin-embedded blocks from which 4-µm-thick sections were cut and stained with H&E to analyze sample viability, i.e., the presence of airways and lung parenchyma, and the absence of infection, cancer, and fibrosis. Lung tissue was fixed in 10% buffered formalin, was routinely processed, and was embedded in paraffin. 

New 4-µm-thick sections of each selected block were cut and deparaffinized. Slides were stained with the following monoclonal antibodies: anti-ACE2 (Thermo Fisher Scientific, Waltham, MA, USA, mouse, Cat# MA5-31395, RRID:AB_2787031, 1:2,500); anti-TMPRSS2 (Abcam, Cambridge, UK, rabbit, Cat# ab109131, RRID:AB_10863728, 1:3,000), and anti-SIRT1 (Santa Cruz Biotechnology, Inc, Santa Cruz, CA, USA, mouse, Cat# sc-135792, RRID:AB_2188481, 1:200). 

### Image analysis and quantification

All slides were scanned with Pannoramic Viewer software, version 1.15.2 for Windows (3DHISTECH, Budapest, Hungary), and image analyses were performed with Image-Pro Plus 4.5 software for Windows (Media Cybernetics, Rockville, MD, USA). 

For the lung parenchyma, 15 regions of interest per patient were randomly chosen and 333-335 µm of alveolar septum were selected at 400× magnification, totaling 5 mm of alveolar septum analyzed per patient for each antibody. For the airways, the epithelial expression of ACE2, TMPRSS2, and SIRT1 in non-cartilaginous and cartilaginous airways was quantified in a length of at least 3 mm basement membrane per airway type, for each age group.

The lengths of the alveolar septum and airway epithelium were manually demarcated with the software drawing tool. We defined a color range considered positive staining for each antibody and applied it to all cases to calculate the positive area. This standard was applied to the selected area, and the positive stained area was calculated. The positive stained area was normalized to the corresponding alveolar septum or airway epithelium length (µm^2^/µm).^23^

### Statistical analysis

Analyses were performed with the IBM SPSS Statistics software package, version 21.0 for Windows (IBM Corporation, Armonk, NY, USA). The data distribution was determined by the Kolmogorov-Smirnov normality test, which showed that our data were not normally distributed. The Mann-Whitney and Kruskal-Wallis tests were therefore used to compare groups. Variables are presented as median and interquartile range. The correlations were analyzed by the Spearman test. Values of p < 0.05 were considered statistically significant. 

## RESULTS

The study population consisted of 43 patients (19 Females and 24 Males). The median age of the 25 children (11 F / 14 M) was 7 years (range 0.03-19); the median age of the 7 adults (2 F / 5 M) was 48 years (range 29-59); and the median age of the 11 elderly individuals (6 F / 5 M) was 78 years (range 60-95). 

The pediatric group mainly presented with malformations, malignancies, and autoimmune diseases as underlying conditions, and the most common causes of death were cerebral edema and heart failure. The adults and elderly patients had diabetes mellitus and systemic arterial hypertension and died from cardiovascular causes. Table 1 shows the age, sex, underlying conditions, and immediate cause of death of each patient in the study population. 

**Table 1:** Age, sex, underlying conditions, and causes of death in the study population.

Case	Age	Sex	Underlying condition	Cause of death
1	10 days	F	Cardiovascular malformations	Congestive heart failure
2	11 days	M	Inborn metabolism error (urea cycle defect)	Multiple organ failure
3	1 month	F	Cardiac rhabdomyoma submitted to chemotherapy	Heart failure
4	1 year	F	Acute myeloid leukemia	Hypovolemic shock by acute gastrointestinal hemorrhage
5	1 year	M	Undifferentiated central nervous system embryonal tumor, probably medulloblastoma	Intracranial hypertension
6	2 years	M	Left intraventricular and intracerebral ependymoma	Cerebral edema
7	2 years	M	Central nervous system high-grade B-cell lymphoma; combined immunodeficiency	Intracranial hypertension
8	3 years	M	Klippel-Trenaunay-Weber syndrome; multiple hemangiomas	Cerebral edema caused by hemorrhage from ruptured hemangiomas
9	3 years	M	Chronic nephropathy (segmental glomerular sclerosis)	Heart failure
10	4 years	F	Takayasu arteritis; dilated cardiomyopathy	Congestive heart failure
11	6 years	F	Stage 4 neuroblastoma with lymph node metastasis	Cardiorespiratory arrest after seizure
12	6 years	M	Myelodysplasia	Intracranial hypertension from stroke
13	7 years	M	Crohn’s disease	Hypovolemic shock caused by gastrointestinal hemorrhage
14	7 years	F	Systemic autoimmune disease	Cardiogenic shock caused by cardiac microinfarctions
15	10 years	M	Undifferentiated malignant small cell tumor of the brain	Cerebral edema
16	10 years	F	Caroli disease; liver transplantation and retransplantation	Liver failure caused by portal vein thrombosis with hypovolemic shock
17	11 years	F	Fulminant autoimmune hepatitis; liver transplantation	Liver failure with hypovolemic shock
18	11 years	M	Meningeal melanoma	Cerebral edema
19	12 years	F	Extrahepatic bile duct atresia with biliary liver cirrhosis; liver transplantation with chronic rejection	Liver failure
20	14 years	M	Cerebral arteriovenous malformation	Distributive shock, cerebral edema, and hemorrhage stroke
21	15 years	F	Operated Frantz tumor (solid pancreatic pseudopapillary tumor); chronic cholestatic hepatopathy	Heart failure; extensive hepatic steatosis
22	17 years	M	SLE; thrombotic thrombocytopenic purpura; ischemic stroke; hypothyroidism; atopic dermatitis	Alveolar hemorrhage; coagulopathy
23	18 years	M	Nasopharyngeal angiofibroma	Hypovolemic shock caused by acute hemorrhage in the operative site
24	18 years	F	Diffuse germinal center B-cell non-Hodgkin lymphoma	Circulatory shock
25	19 years	M	Pilocytic astrocytoma; intracranial hemorrhage	Cerebral edema
26	29 years	M	Alcohol abuse; chronic liver disease	Acute pancreatitis
27	38 years	M	No comorbidities	Hemopericardium (acute aortic dissection)
28	46 years	M	SAH; hepatic cirrhosis from alcohol abuse	Hepatic failure
29	48 years	M	Alcohol abuse	Heart failure
30	49 years	F	Hypertensive heart disease	Heart failure
31	58 years	F	Heart disease	Heart failure
32	59 years	M	DM; SAH; alcohol abuse; Guillain-Barré syndrome	Acute myocardial infarction
33	60 years	M	DM; SAH	Acute myocardial infarction
34	62 years	M	DM; SAH	Heart failure
35	66 years	F	DM; SAH; stroke	Ischemic stroke
36	73 years	M	DM; SAH	Acute myocardial infarction
37	76 years	F	DM; SAH; heart disease	Heart failure
38	78 years	M	DM; SAH	Congestive heart failure
39	81 years	F	DM; SAH; dementia	Acute pyelonephritis
40	87 years	M	DM; SAH; heart disease; neurological disorder	Acute myocardial infarction
41	87 years	F	Advanced duodenal carcinoma	Cachexia
42	88 years	F	DM; SAH; multiple myeloma	Ischemic heart disease
43	95 years	F	Dementia	Pulmonary thromboembolism

F: female; M: male; SLE: systemic lupus erythematosus; DM: diabetes mellitus; SAH: Systemic Arterial Hypertension.

**Figure 1:**
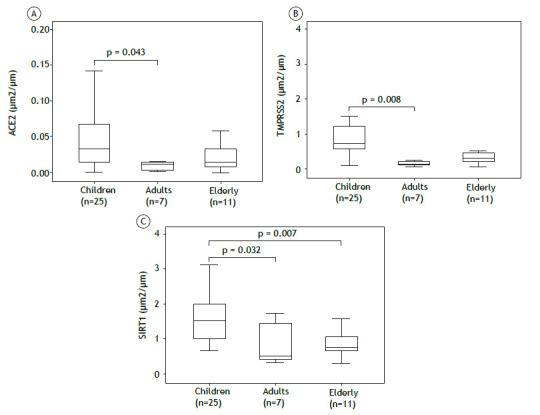
Expression of ACE2 (A), TMPRSS2 (B), and SIRT1 (C) in the lung parenchyma of children (0- to 19-years old), adults (20- to 59-years old), and elderly individuals (≥ 60 years old). The results are presented as median [IQR]. Data were obtained as a ratio of immunostained positive area to alveolar septum length (µm^2^/µm) in the lung parenchyma and as a ratio of immunostained positive area to epithelial basal membrane length (µm^2^/µm) in the airways. ACE2, Angiotensin-Converting Enzyme 2; TMPRSS2, Transmembrane Serine Protease 2; SIRT1, Sirtuin 1.

**Figure 2:**
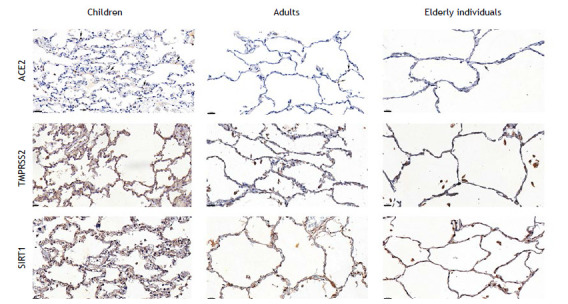
Expression of ACE2, TMPRSS2, and SIRT1 in the alveolar walls of individuals of different ages. Children have increased ACE2, TMPRSS2, and SIRT1 protein expression.

ACE2, TMPRSS2, and SIRT1 protein expression was greater in the alveolar parenchyma of children (p = 0.043, p = 0.008, and p = 0.032, respectively) than in that of adults (Table 2, Figures 1 and 2). In addition, SIRT1 was expressed more in the lung parenchyma of children than in that of elderly individuals (p = 0.007) (Table 2, Figure 1, and Figure 2). There was no statistically significant difference in the expression of ACE2, TMPRSS2, or SIRT1 in the airways among children, adults, and elderly patients (Table 2). 

**Table 2:** Descriptive analysis of ACE2, TMPRSS2 and SIRT 1 expression in the lung tissues of children (0- to 19-years old), adults (20- to 59-years old), and elderly individuals (≥ 60 years old).^a^

Structure	Markers	n	Children	n	Adults	n	Elderly individuals	p
Lung parenchyma	ACE2	25	0.032 [0.015 - 0.067]*	7	0.012 [0.003 - 0.015]	11	0.015 [0.005 - 0.035]	0.030
	TMPRSS2	25	0.745 [0.596 - 1.235]*	7	0.144 [0.120 - 0.274]	11	0.331 [0.167 - 0.520]	0.003
	SIRT1	24	1.586 [1.036 - 2.023]*	7	0.581 [0.408 - 1.641]	11	0.815 [0.700 - 1.243]**	0.002
	ACE2/ TMPRSS2	25	0.032 [0.017 - 0.135]	7	0.089 [0.008 - 0.097]	11	0.049 [0.021 - 0.162]	0.963
Airways	ACE2	19	0.021 [0.010 - 0.054]	5	0.005 [0.001 - 0.012]	6	0.014 [0.004 - 0.021]	0.064
	TMPRSS2	19	5.057 [2.984 - 9.757]	5	2.799 [1.200 - 5.043]	6	3.376 [0.696 - 4.289]	0.160
	SIRT1	25	8.540 [0.000 - 14.520]	7	5.840 [0.000 - 8.570]	11	4.120 [0.000 - 6.290]	0.060
	ACE2/ TMPRSS2	19	0.005 [0.002 - 0.011]	5	0.001 [0.001 - 0.002]	6	0.006 [0.001 - 0.014]	0.283

ACE2: angiotensin-converting enzyme 2; TMPRSS2: transmembrane serine protease 2; and SIRT1: sirtuin 1. ^a^Data presented as median [IQR] and obtained as a ratio of immunostained positive area to alveolar septum length (µm^2^/µm) in the lung parenchyma and as a ratio of immunostained positive area to epithelial basal membrane length (µm^2^/µm) in the airways. *Children ≠ adults: ACE2, p = 0.043; TMPRSS2, p = 0.008; and SIRT1, p = 0.032. **Children ≠ elderly individuals: SIRT1, p = 0.007.

There was no significant difference in ACE2, TMPRSS2, or SIRT1 protein expression between female and male patients in the pulmonary parenchyma or the airways (supplementary material, Table S1). There was no significant difference in ACE2, TMPRSS2, or SIRT1 in the lungs of children when stratified by age (supplementary material, Table S2). 

**Figure 3:**
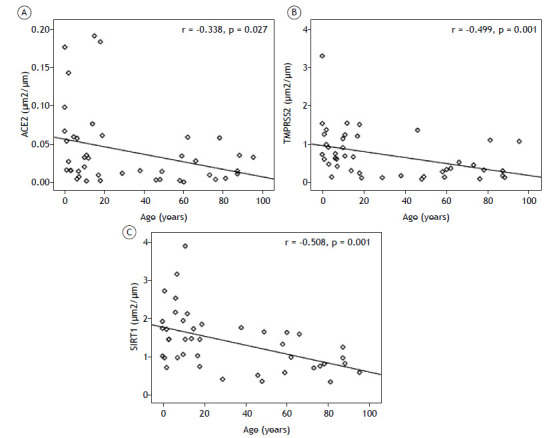
Correlations between age and expression of ACE2 (A), TMPRSS2 (B), and SIRT1 (C) in the pulmonary parenchyma. The results are presented as median [IQR]. Data were obtained as a ratio of immunostained positive area to alveolar septum length (µm^2^/µm) in the lung parenchyma and as a ratio of immunostained positive area to epithelial basal membrane length (µm^2^/µm) in the airways. ACE2, Angiotensin-Converter Enzyme 2; TMPRSS2, Transmembrane Serine Protease 2; SIRT1, Sirtuin 1.

There were negative correlations between age and expression of ACE2 (r = −0.338; p = 0.027), TMPRSS2 (r = −0.499; p = 0.001), and SIRT1 (r = −0.508; p = 0.001) in the pulmonary parenchyma (Figure 3). There were no significant correlations between ACE2, TMPRSS2, and SIRT1 protein levels in the lung compartments. 

## DISCUSSION

In the present study, we investigated the expression of ACE2, TMPRSS2, and SIRT1 proteins in the lungs of deceased individuals without any pulmonary disease, with normal lung histology, comprising the entire human age spectrum. Our results showed that, in the pulmonary parenchyma, these proteins were more expressed in children than in adults, and SIRT1 was more expressed in the pediatric group than in the alveoli of elderly patients. There were no differences in protein expression in the airway epithelium. Additionally, there was no significant difference between females and males or among children separated in different age groups. 

Our study is the first to use a semiautomated quantitative method to analyze these proteins and corroborated findings that not only ACE2 but also TMPRSS2 and SIRT1 are more expressed in the lung parenchyma of children. In older individuals, the lower ACE2 levels, by amplifying angiotensin II action, supports pathological processes such as endothelial dysfunction, thrombosis, deleterious inflammation, oxidative stress, and vasoconstriction.^1^ When the SARS-CoV-2 envelope fuses with a cell membrane, the extracellular component of ACE2 is internalized and inactivated, thus downregulating this protein.^24^ Although counterintuitive, while acting as a receptor for virus entry, the higher expression of membrane-bound ACE2 in alveolar epithelial cells could also protect against RAS system imbalance,^25^ which could contribute to the worsening of lung injury. 

Viral pneumonia is one of the causes of severe ARDS, and imbalance of the RAS system is part of the pathophysiology of ARDS. Accordingly, ACE2 knockout mice develop more severe ARDS when subjected to lung injury caused by acid^26^ ^,^ ^27^ and when infected with respiratory syncytial virus.^28^ It has been suggested that the upregulation of ACE2 could reduce the ARDS-related lung injuries caused by RAS dysregulation.^26^ ^,^ ^29^

Factors other than ACE2 and TMPRSS2 expressions are also likely to play a significant role in protecting children against more severe COVID-19. Among these factors, greater effectiveness of nasal mucosal innate immunity against SARS-CoV-2 in children^30^ could prevent the arrival of a significant amount of virus deep in the lungs, which has been associated with disease severity. Hofstätter et al.^31^ also suggested that the respiratory physiology of children provides further protection against effective aerosol SARS-CoV-2 inoculation in the lower respiratory tract. 

Interestingly, in the airways there was no difference in ACE2, TMPRSS2, or SIRT1 expression, suggesting that these proteins in these lung structures play no age-related role in possibly defending against or facilitating infection by viruses such as SARS-CoV-2. 

One of the initial hypotheses for COVID-19 being frequently more severe in adults than in children was that the latter could express less ACE2 and TMPRSS2 in the respiratory tract than older individuals.^11^ ^,^ ^12^ This was not confirmed in our results. Nevertheless, studies have shown conflicting results (Table 3).^16^ ^-^ ^19^

**Table 3:** Comparison of ACE2 protein expression across studies.

Features	Schurink et al.^16^	Silva et al.^17^	Ortiz et al.^18^	Zhang et al.^19^	Present study
Lung tissue obtained from	Autopsy	Lung segmentectomy or lobectomy	Donor lungs	Lung segmentectomy or lobectomy	Autopsy
Lung compartment analyzed	Alveoli and airways	Alveoli (alveolar type II cells)	Alveoli and airways	Alveoli and airways	Alveoli and airways
Number of cases	20	76	14	50	43
Age groups	15 children (24w+3d to 18y), 5 young adults (23-28y)	21 children (1-18y), 26 adults (20-59y), 29 elderly individuals (≥60y)	6 children (0.5-5y), 6 adults (26-57y), 2 elderly individuals (64y, 71y)	26 children (0.16-12y), 14 adults (16-49y), 10 elderly individuals (50-80y)	25 children (0.03-19y), 7 adults (20-59y), 11 elderly individuals (60-95y)
Method	Qualitative (immunostaining intensity by 2 pathologists)	Semi-quantitative (positive alveolar type II cells staining intensity classified from +1 to +3)	Semi-quantitative (% positive cells: 0 = no detection 1 = rare < 1% 2 = 1-33% 3 = 34-66% 4 = > 66%)	Semi-quantitative (number of positive cells per 0.025 cm² of lung tissue)	Quantitative (Protein expression assessed with software)
Age-related changes	ACE2 ­ (Reached a plateau at 5y)	ACE2­ and TMPRSS2 ≈	ACE2 ¯	ACE2 ¯	ACE2¯, TMPRSS2¯, and SIRT1¯

w: weeks of age; d: days of age; y: years of age; ­: increased; ≈: similar; and ¯: decreased.

Schurink et al.^16^ qualitatively evaluated ACE2 immunostaining intensity in the alveoli and airways of lungs from 15 children (from fetuses to adolescents) and 5 young adults and showed that ACE2 expression was lower in younger children and increased until reaching a plateau at the age of 5 years. Silva et al.^17^ analyzed the percentage of ACE2 and TMPRSS2 staining positivity in alveolar type II cells in the lung parenchyma of 21 children, 26 adults, and 29 elderly individuals, classifying the staining intensity from +1 to +3. The study revealed that ACE2 was less expressed in the alveoli of children than in those of adults and elderly individuals, and TMPRSS2 was not different among age groups.^17^ In contrast, Ortiz et al.^18^ graded the percentage of lung ACE2-positive cells and showed that this protein was more expressed in the lungs of 6 children than in those of 8 adults/elderly patients. Zhang et al.^19^ quantified the number of ACE2-positive cells per 0.025 cm^2^ of lung tissue in 26 children under 12 years of age and 24 adults/elderly individuals. This protein was more expressed in the lungs of children and declined progressively when the samples were grouped as 0-10 years of age, 10-50 years of age, 50-60 years of age, 60-70 years of age, and > 70 years of age, with no difference between the first two groups.^19^

We detected increased levels of TMPRSS2 in the lung parenchyma of children. Although TMPRSS2 also acts as a primer for human metapneumovirus^32^ and human parainfluenza virus,^33^ viruses responsible for important respiratory infections, there are limited data on this protein expression in lung tissue. Regarding COVID-19 severity, TMPRSS2 activity was supposed to assist explaining the differences between sexes,^34^ but we found no differences between female and male individuals in our results. 

The present study is the first to analyze SIRT1 in the lungs of children. We found that SIRT1 was more expressed in children than in adults and elderly individuals. This sirtuin amplifies ACE2 transcription and protein expression and is upregulated under energy stress,^22^ as occurs in ARDS. Furthermore, there are several pathways in which sirtuins appear to participate in host defense against viral infection, including against SARS-CoV-2 disease. Sirtuins are known to upregulate nuclear factor erythroid 2-related factor 2 (NRF2), a molecule with important antioxidant properties,^35^ and other studies have shown decreased levels of NRF2 in children during viral respiratory infections.^36^ ^,^ ^37^ Sirtuins also have antiviral and anti-inflammatory effects by promoting cell autophagy and inhibiting the activation of the nucleotide-binding domain, leucine-rich repeat, and pyrin domain-containing protein 3 (NLPR3) inflammasome.^38^ The SARS-CoV-2 nonstructural protein 14 (NSP14) interacts with SIRT1, inhibiting its ability to activate the NRF2/HMOX1 pathway.^35^ It is likely that the increased levels of SIRT1 in the lung parenchyma of children may be another protective factor in modulating COVID-19 severity. 

Our study has several limitations. First, the small sample size prevented us from addressing all possible confounding factors, such as the underlying diseases. However, it is particularly challenging to find near normal lung tissue obtained from autopsy. Previous studies have also reported a limited number of samples, reinforcing the difficulty in obtaining nondiseased tissue.^16^ ^,^ ^18^

Second, a decay in antigenicity through time can be observed in human tissue embedded in paraffin.^39^ In our study, the lung tissue from children had been preserved in paraffin for longer periods than had those from adults and elderly individuals. However, since we described an increased expression of ACE2, TMPRSS2, and SIRT1 in children, a possible decrease in antigenicity would have even strengthened our results. In addition, tissue samples from our autopsy archives are preserved by rigorous control of humidity and temperature, and we used antigen retrieval methods during immunohistochemical staining to address the issue of antigen decay. 

Third, we have not studied COVID-19 cases for this research, because fortunately we did not have a considerable number of deaths from COVID-19 pneumonia among children in our hospital. Although it might be difficult to compare our data with those from other studies, we quantitatively analyzed ACE2, TMPRSS2, and SIRT1 proteins in individuals of a wide age range, and this may have led to more robust results. 

In summary, our study analyzed the entire human age spectrum and showed that children without pulmonary disease have more membrane-bound ACE2, TMPRSS2, and SIRT1 protein levels in the alveoli. It is likely that the putative protective effects of ACE2 and SIRT1 against lung injury, together with a more competent innate immune system in the upper airways, may have fortunately contributed to less severe COVID-19 lung disease in children, even in the prevaccine era. 
